# Acute injury to the mouse carotid artery provokes a distinct healing response

**DOI:** 10.3389/fphys.2023.1125864

**Published:** 2023-02-07

**Authors:** Timothy Warwick, Giulia Karolin Buchmann, Beatrice Pflüger-Müller, Manuela Spaeth, Christoph Schürmann, Wesley Abplanalp, Lukas Tombor, David John, Andreas Weigert, Martin Leo-Hansmann, Stefanie Dimmeler, Ralf P. Brandes

**Affiliations:** ^1^ Institute for Cardiovascular Physiology, Goethe University Frankfurt, Frankfurt am Main, Germany; ^2^ German Center for Cardiovascular Research (DZHK), Partner site Rhein Main, Frankfurt am Main, Germany; ^3^ Institute of Cardiovascular Regeneration, Goethe University Frankfurt, Frankfurt am Main, Germany; ^4^ Institute of Biochemistry I, Faculty of Medicine, Goethe University Frankfurt, Frankfurt am Main, Germany; ^5^ Department of Pathology, University Hospital Frankfurt, Frankfurt am Main, Germany

**Keywords:** smooth muscle cells, phenotypic switching, vascular injury, single-cell, healing, neointima, inflammation, transcriptomics

## Abstract

Treatment of vascular stenosis with angioplasty results in acute vascular damage, which may lead to restenosis. Owing to the highly complex cellularity of blood vessels, the healing response following this damage is incompletely understood. To gain further insight into this process, scRNA-seq of mouse carotid tissue after wire injury was performed. Stages of acute inflammation, resolution and remodeling were recapitulated in these data. To identify cell types which give rise to neointima, analyses focused on smooth muscle cell and fibroblast populations, and included data integration with scRNA-seq data from myocardial infarction and atherosclerosis datasets. Following carotid injury, a subpopulation of smooth muscle cells which also arises during atherosclerosis and myocardial infarction was identified. So-called stem cell/endothelial cell/monocyte (SEM) cells are candidates for repopulating injured vessels, and were amongst the most proliferative cell clusters following wire-injury of the carotid artery. Importantly, SEM cells exhibit specific transcriptional profiles which could be therapeutically targeted. SEM cell gene expression patterns could also be detected in bulk RNA-sequencing of neointimal tissue isolated from injured carotid vessels by laser capture microdissection. These data indicate that phenotypic plasticity of smooth muscle cells is highly important to the progression of lumen loss following acute carotid injury. Interference with SEM cell formation could be an innovative approach to combat development of restenosis.

## 1 Introduction

Angioplasty and stenting are routine clinical interventions to treat vascular stenosis. While promoting early lumen gain, both procedures also impose severe and acute vascular injury (Wu et al., 2017), and consequently late lumen loss and restenosis. Restenosis is mediated by neointima development and fibrotic scarring of the vessel (Smith et al., 2019). Clinically, the problem of post-angioplasty restenosis has been partially solved by drug-eluting balloons and stents (Moses et al., 2003). However, given that these approaches also limit endothelial regeneration, they remain suboptimal. During angioplasty, local tissue destruction provokes the release of damage-associated molecules and plaque debris, which results in local inflammation. Subsequently, all cell types of the vessel (endothelial cells, fibroblasts and smooth muscle cells) proliferate (Szöcs et al., 2002), a process indispensable for vascular repair (Owens et al., 2004; Gallo et al., 2018). Induction of proliferation requires profound reprogramming of cells. For example, smooth muscle cells (SMC) exist in a highly differentiated state in healthy vessels, allowing them to control vascular tone. Upon injury, transdifferentiation towards a proliferative, migratory and secretory phenotype occurs, albeit at the expense of a downregulation of the contractile machinery.

Technical limitations have made the study of individual cell state and associated cellular dynamics difficult. With the advent of single-cell RNA-sequencing (scRNA-seq), it is now possible to profile gene expression on the level of individual cells in an unbiased manner (Dobnikar et al., 2018). This technique has therefore revolutionized the analysis of dynamic processes in complex tissues. Cellular dynamics in atherosclerosis (Pan et al., 2020), myocardial infarction (Tombor et al., 2021) and vascular aneurysms (Yang et al., 2021) have subsequently been reported on at single-cell resolution, with additional studies being reported increasingly often. This large body of literature and accessible data has helped to identify individual cell types involved in processes relevant to cardiovascular disease. Moreover, it facilitates comparative analyses between different vascular disease states, and therefore the identification of common and unique cell populations and their crosstalk. Spatial transcriptomics goes further by mapping transcriptional profiles onto histological sections (Rao et al., 2021), but resolution of the technique is still limited. Spatial transcriptomics has been applied to material derived from myocardial infarction (Kuppe et al., 2022), but data generated from the vasculature is currently lacking.

In the present study, we analysed the response of the murine carotid artery to wire injury, using time-resolved scRNA-seq and bulk RNA-seq of neointimal tissue isolated by laser capture microdissection. Importantly, these experiments were performed in the absence of a high-fat diet, permitting sole focus on the naive injury response. This choice was made due to the wide-scale bias towards atherogenic diets in vascular studies. Considering the high lipid and cholesterol content of these diets, and the frequent addition of pro-inflammatory agents like cholate, this dietary intervention has a complex impact on immunity. In fact, these diets have been developed to massively accelerate atherosclerosis development (Daugherty et al., 2017), but ignore that human atherosclerosis is a slow process in which inflammation is just one of many risk factors and drivers. There is no doubt, that inflammation also contributes to atherosclerosis development in humans. However, considering the long duration of the disease and its different phases, precise understanding on the contribution of inflammation is still emerging (Soehnlein and Libby, 2021).

## 2 Materials and methods

### 2.1 Mouse studies and carotid injury model

The present study consists of two datasets: One study on the naive carotid artery and one dataset on restenosis. For the analysis of the healthy vessels, cell isolation procedures were partially adapted from Hu et al. (2016) to ensure isolation of *bona fide* carotid cell populations, rather than cells of the neointima or granulation tissue. When analyzing the injury response of the murine carotid artery, the entire vascular cell population was of interest. Data subset from the latter dataset have been used in a previous study comparing the vascular response in WT and Nox4^−/−^ mice (Buchmann et al., 2021).

All animal experiments were performed in accordance with the National Institutes of Health Guidelines on the use of laboratory animals. The University Animal Care Committee and the Federal Authorities for Animal Research (Darmstadt, Germany) approved the study protocol (approval number: FU1185). Mice were housed in a specified pathogen-free facility with 12-12 h day and night cycle and free access to water and chow. Wire-induced injury surgery was applied to male mice at an age of 11 weeks. Wire-injury was carried out only on the left carotid of the animal, the right carotid artery served as control (Lindner et al., 1993; Xu, 2004; Bigalke et al., 2011). Due to the potential for artefacts and variability, any vessels showing signs of thrombosis were removed and not subjected to further analysis.

### 2.2 Histology

Carotid tissue samples frozen in OCT compound were prepared as 10 µm serial sections. Sections were stained with Hematoxylin and Eosin staining solutions. The neointima area was analyzed by planimetry with ImageJ.

### 2.3 Single cell RNA sequencing

The experiments were conducted as reported previously (Buchmann et al., 2021), with modifications.

The following adapted protocol (Hu et al., 2016) was used to obtain single cells from the healthy murine carotid artery: Eight carotid arteries from male mice were cut into small pieces in an enzyme mixture containing 125 U/mL collagenase type XI, 450 U/mL collagenase type I, 60 U/mL hyaluronidase type I-s and 60 U/mL DNase one in PBS containing Ca^2+^ and 20 mM HEPES (pH. 7.4) and subsequently incubated at 37 °C for 30 min in a total volume of 5 mL. Every 10 min, the solution was resuspended by pipetting. Digestion was stopped with stopping solution (PBS; 2% FCS; 1 mM EDTA) and the cell suspension was strained.

For the wire injury samples, a minimum of five carotid arteries were used per time point following wire injury, each originating from a male mouse. Any vessels showing signs of thrombosis were removed from the experiments, in order to avoid variability and potential artefacts. Carotids were cut in small pieces and digested for 1 h at 37°C with an enzyme mix containing collagenase type XI (125 U/mL), collagenase type I (450 U/mL), hyaluronidase (60 U/mL) and deoxyribonuclease I (60 U/mL). Digestion was stopped with stopping solution (PBS; 2% FCS; 1 mM EDTA) and the cell suspension was strained. Dead cells were removed by MACS Dead cell removal kit (Miltenyi, Germany). Cells were washed and resuspended in PBS and used for droplet scRNA-seq.

For both datasets, suspensions were loaded on a 10x Chromium Controller (10x Genomics) according to manufacturer’s protocol. All scRNA-seq libraries were prepared using Chromium Single Cell 3 v2 Reagent Kit (10x Genomics) according to manufacturer’s protocol. In brief, the initial step consisted of producing an emulsion where individual cells were isolated into droplets together with gel beads coated with unique primers bearing 10x cell barcodes, UMI (unique molecular identifiers) and poly(dT) sequences. Reverse transcription reactions were engaged to generate barcoded full-length cDNA, followed by the disruption of emulsions using the recovery agent and cDNA clean up with DynaBeads MyOne Silane Beads (Thermo Fisher Scientific, Germany). Bulk cDNA was amplified using a Biometra Thermocycler TProfessional Basic Gradient with 96-well Sample Block (98 °C for 3 min; cycled 14 times: 98 °C for 15 s, 67 °C for 20 s, and 72 °C for 1 min; 72 °C for 1 min; held at 4 °C). Amplified cDNA product was cleaned with the SPRIselect Reagent Kit (Beckman Coulter, United States). Indexed sequencing libraries were constructed using the reagents from the Chromium Single Cell 3 v2 Reagent Kit, as follows: fragmentation, end repair and A-tailing; size selection with SPRIselect; adaptor ligation; post-ligation cleanup with SPRIselect; sample index PCR and cleanup with SPRI select beads. Library quantification and quality assessment were performed using Bioanalyzer Agilent 2,100 using a High Sensitivity DNA chip (Agilent Genomics, United States). Indexed libraries were equimolarly pooled and sequenced on two Illumina HiSeq4000 using paired-end 26 × 98 bp as sequencing mode by GenomeScan (Leiden, Netherlands).

### 2.4 Single-cell RNA-sequencing data analysis

Single-cell expression data were processed using the Cell Ranger Single Cell Software Suite (v2.1.1) to perform quality control, sample de-multiplexing, barcode processing, and single-cell 30 gene counting. Sequencing reads were aligned to the mouse reference genome GRCh38 using the Cell Ranger suite with default parameters. Data transformation and dimensionality reduction was carried out using the *Seurat* (v4.1.1, Hao et al. (2021)) package for *R*. During transformation, the percentage of mitochondrial reads mapped per cell was regressed out.

Data for integration was accessed as follows. ScRNA-seq data from cardiac tissue from Tombor et al. (2021) was available from ArrayExpress (accessions E-MTAB9816 and E-MTAB9817). Data from aortic tissue (Pan et al., 2020) was accesed *via* NCBI Gene Expression Omnibus (GEO) under the series GSE155513. Each dataset was processed identically prior to integration. The integration itself was performed using *Seurat*, after identification of variable features and subsequently integration anchors.

Marker genes of cell clusters determined by *Seurat* were identified using the *FindAllMarkers* function in conjunction with the *blmod* test. Marker genes were those with *Padj* < 0.05. Cell type marker genes were taken from literature and curated databases (Franzén et al., 2019; Shao et al., 2020), and used to assign likely cell types to clusters. These assignments were then used in subsequent analyses of cell proportions, dynamics and cell cycle scoring comparisons. For cell cycle scoring, genes for classification of S and G2/M phases in *Mus musculus* were accessed at https://github.com/hbc/tinyatlas/tree/master/cell_cycle. Pathway enrichment analyses were carried out using the *R* package *gprofiler2* (Kolberg et al., 2020).

Single-cell trajectories were constructed using the *R* package *Monocle 3* (v1.3.1) (Cao et al., 2019), with a clustering resolution of *k* = 40.

### 2.5 Laser capture microdissection

Frozen tissue sections were mounted on special LCM membrane slides and stained immunohistochemically with Hematoxylin and Eosin to detect carotids and elastic laminas. Carotid sections containing outer elastic lamina and neointima were then cut and catapulted into the lid of a collection tube by the laser. After collection of up to 70 sections, total RNA was extracted using an RNeasy Micro Kit (Qiagen, Hilden, Germany). nanoMACE-seq was performed for further analysis.

### 2.6 NanoMACE-seq and bioinformatics

The library preparation was conducted by GenXPro (Frankfurt, Germany). 3′mRNA libraries were prepared using GenXPros “Rapid MACE-Seq Kit” for low-input mRNA according to the manual of the manufacturers. Briefly, RNA was fragmented to an average size of 350 bps, followed by poly-A specific cDNA synthesis, pooling and amplification by PCR using the minimum number of cycles as described in the manual. The PCR product was purified by SPRI purification. The final product of 200–400 bps was quality controlled on a Perkin Elmer LabChip GXII, the concentration was measured on Qbit. Sequencing was performed on a Illumina NextSeq500 machine with 1x 75 bps. The MACE-reads were demultiplexed according to the sample IDs, PCR duplicates were removed with the help of the “TrueQuant” UMI barcodes. Low-quality and adapter-containing reads were cropped. The reads were quantified against the *mm39* transcriptome using *Salmon* (v1.9.0, Patro et al. (2017)). Differential gene expression of the different pairwise comparisons was analyzed using DESeq2 (v1.36.0, Love et al. (2014)). The normalized gene counts, *p*-values and log2fold-change values of all pairwise comparisons were combined in a single table. Differentially expressed genes were used as input to gene set enrichment analysis which was computes using the *gprofiler2* package for *R* (Kolberg et al., 2020; R Core Team, 2022).

Gene-trait associations were checked using the NHGRI-EBI GWAS Catalogue (Buniello et al., 2019). Human homologues of mouse genes were used to query the database for significantly linked traits.

## 3 Results

### 3.1 The murine carotid artery is rich in smooth muscle cells

**FIGURE 1:**
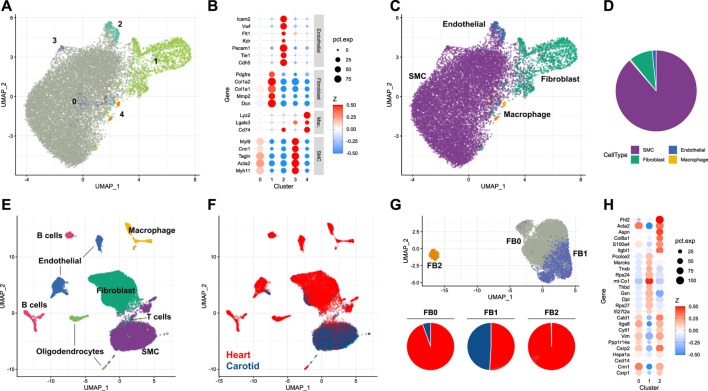
Single-cell analysis of the naive murine carotid artery **(A)** Uniform manifold approximation projection (UMAP) plot of cells isolated from homeostatic carotid arteries, with clusters assigned by *Seurat*. **(B)** Scaled expression of cell-type marker genes in each cell cluster. **(C)** UMAP plot of cells coloured by assigned cell type. **(D)** Proportions of cells assigned as smooth muscle cells (SMC), fibroblasts, endothelial cells and macrophages. **(E, F)** Integration of single-cell RNA-sequencing data from carotid and cardiac tissues. **(G)** Re-clustering of integrated fibroblasts, and proportions of each cluster originating from either carotid or cardiac tissue. **(H)** Cluster marker genes for each integrated fibroblast cluster.

To understand the dynamics of the carotid artery post-injury, a characterisation of the cellular landscape of the naive, uninjured vessel was required. This was obtained by using an adapted digestion protocol for isolating single cells from eight carotid arteries of male mice (see *Materials and methods*). This was particularly suited to the harvesting of differentiated smooth muscle cells residing in the tunica media. Cells isolated with this approach were then subjected to scRNA-seq. 15,285 cells were subjected to dimensionality reduction and cluster assignment (Figure 1A), and gene expression patterns of resulting clusters (Figure 1B) were used to assign cell types (Figure 1C **)**. The most abundant cell type identified in the naive carotid artery were smooth muscle cells, which made up 88.6% of the total number of cells. The second-most abundant cell population were fibroblasts (9.3%), followed by endothelial cells (1.6%) and finally macrophages (0.5%) (Figure 1D). To check how carotid cell composition contrasts to aortic and cardiac tissues, the proportions of each cell type in uninjured tissue were compared. Carotid and aortic (Pan et al., 2020) tissues were both dominated by smooth muscle cells, which made up more than 50% of total cells in each dataset. In uninjured cardiac tissue (Tombor et al., 2021), it was fibroblasts which were the most numerous cell type **(** Supplementary Figure S1 **)**. Given that fibroblast heterogeneity has been reported to play a key role in wound healing and repair (Talbott et al., 2022), the carotid fibroblast population was further analyzed.

### 3.2 Carotid fibroblasts show divergent heterogeneity to cardiac fibroblasts

Depending on the organ in question, fibroblasts reside in different niches. In the carotid artery, fibroblasts are predominantly located in the adventitial compartment, i.e. the paravascular niche, whereas fibroblasts of the tunica muscularis are expected to be rarer. Contrastingly, interstitial fibroblasts are frequent in some organs, particularly the heart. Fibroblast heterogeneity is well-established in cardiac tissue, and has been linked to key cardiac functions, including response to injury (Wang et al., 2022). To examine whether similar fibroblast heterogeneity exists and might be of functional importance in the carotid artery, the carotid dataset was integrated with scRNA-seq data obtained from the non-cardiomyocyte population of healthy mouse hearts (Tombor et al., 2021). When integrating single-cells arising from carotid and cardiac tissue (Figures 1E, F), it became clear that the majority of smooth muscle cells originated from the carotid artery, compared to the fibroblast population which was dominated by cardiac cells. Fibroblast populations were re-clustered and could be split into three distinct groups **(** Figure 1G, UPPER). The most abundant of these populations (FB0, 4,801 cells, 62.7% of total fibroblasts) was dominated by cardiac fibroblasts (Figure 1G, lower), which made up 94.5% of the cluster. Cells assigned to this cluster exhibited gene expression patterns typical of myofibroblasts (Xie et al., 2018) (Figure 1H), including expression of *Acta2*, *Cnn1* and *Vim*. The second-most abundant population (FB1, 2,508 cells, 32.7% of total) was shared between carotid and cardiac datasets. Proportionally, most carotid fibroblasts were assigned to this cluster (82.4% of total carotid fibroblasts). The FB1 cluster was notable for its expression of genes such as *Gsn*, *Dpt* and *Thbd*. This expression profile identifies the cells as representing a subtype of matrix fibroblasts (Wolbert et al., 2020). A third fibroblast cluster (FB2) was restricted almost exclusively to cardiac cells, and was notable for expression of *S100a4*, *Col8a1* and *Fhl2*. These marker genes have previously been attributed to activated fibroblasts (Fei et al., 2017), a class of cells linked heavily to wound healing (Mahmoudi et al., 2019). The absence of this fibroblast subtype in the homeostatic carotid artery indicated that smooth muscle cells, rather than fibroblasts, could also be of importance to the injury response in this tissue.

### 3.3 Carotid injury provokes expansion and dedifferentiation of smooth muscle cells

**FIGURE 2:**
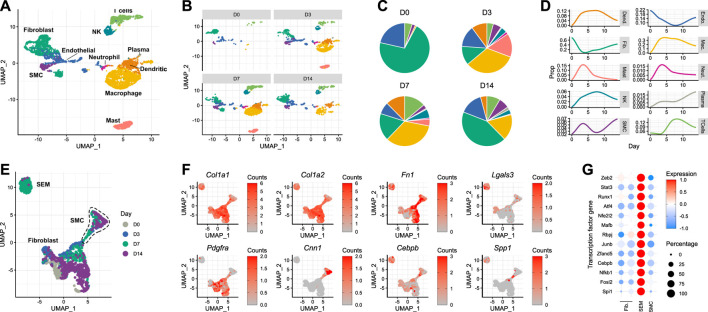
Response of the murine carotid artery to wire injury at single-cell resolution **(A)** UMAP plot of cells isolated from murine carotid arteries on days 0 (uninjured), 3, 7 and 14 after wire injury. **(B)** Cells isolated from carotid arteries, stratified by time point post-injury. **(C)** Cell type proportions at each time point post-injury, colour-matched to UMAP plots. **(D)** Time-resolved changes in cell type proportions. **(E)** Re-clustering of non-immune cells identified in carotid tissue before and after acute injury. **(F)** Gene expression of classical fibroblast (*Pdgfra*) and smooth muscle cell (*Cnn1*) marker genes, as well as reported marker genes for stem cells/endothelial cells/monocyte (SEM) cells. **(G)** Scaled expression of genes encoding transcription factors in non-immune cell types.

Delineating the response of the carotid artery to acute injury required the generation of a time-resolved scRNA-seq dataset from carotid tissue isolated at different time points following wire injury. Carotid arteries were analysed at days 3, 7 and 14 after injury, and compared with the pre-injury state. A minimum of five carotid arteries from male mice were used for each time point. After dimensionality reduction and cell type assignment (Figure 2A), stratification of the data by time point (Figures 2B, C) showed an expected increase in immune cells, whose infiltration into sites of cardiac and vascular damage has been well-characterised (Rurik et al., 2021). Such an infiltration of immune cells would be expected to decrease the relative proportions of tissue-resident carotid cell types described in the previous section. This was true for fibroblast and endothelial populations (Figure 2D), with the latter being directly affected by the mode of injury. However, the same did not apply to smooth muscle cells, which instead underwent an initial expansion in cell proportion in the 3 days following carotid injury. The reason behind this is unclear, and it should be considered that digestion protocols may impose a bias on scRNA-seq data. In this instance, digestion was identical between the time points, meaning comparisons could be made in a cell-type dependent manner.

After day 3, the smooth muscle cell population also began to decrease as a proportion of total cells. Upon closer inspection, a cluster originally annotated as fibroblasts appeared on day 7 post-injury (Figure 2B, bottom left). This cluster was closely-related to the expanding smooth muscle cell population observed on day 3. Although the lack of lineage tracing makes it impossible to determine the origin of these cells, this observation raised the possibility that this population was a form of modulated smooth muscle cell. Indeed, when trajectory analysis was performed on the data, a connection was detected between the smooth muscle cells and this expanding cell population (Supplementary Figure S2). Upon re-clustering of the non-immune cells present in the dataset (Figure 2E), it became clear that the population was distinct from both fibroblasts and smooth muscle cells. The gene expression pattern of the cells in this cluster revealed that they did not necessarily express classical fibroblast (*Pdgfra*) or smooth muscle cell (*Cnn1*) marker genes (Figure 2F). Instead, they exhibited a transcriptional profile which has previously been reported as that of an intermediate cell type derived from smooth muscle cells, termed SEM (stem cell, endothelial cell, monocyte) cells (Pan et al., 2020). These cells were originally named due to their expression of canonical marker genes of each of these 3 cell types. The transcriptional changes described by Pan et al. (2020) in atherosclerotic lesions were evident in the carotid cell population observed here, with expression of *Col1a1*, *Col1a2*, *Fn1*, *Lgals3* and *Cebpb* (Figure 2F). Interestingly, when checking for trait associations (Buniello et al., 2019) of human homologues of mouse SEM cell marker genes, blood pressure and electrocardiogram morphology were top hits, alongside immune cell counts (Supplementary Figure S3). Accompanying these SEM cell marker genes were a number of transcription factor genes, whose expression was markedly increased in the SEM cluster relative to the fibroblast and smooth muscle cell populations (Figure 2G). Amongst them were *Stat3* and *Runx1*, which both encode transcription factors whose functions include maintenance of proliferative capacity and pluripotency (Sherry et al., 2009; Kim et al., 2014). The previously reported behaviours and expression profiles of SEM cells made them interesting candidates for repopulating the carotid artery after acute damage.

### 3.4 Acute and chronic cardiovascular insults result in divergent modulation of smooth muscle cell phenotypes

**FIGURE 3:**
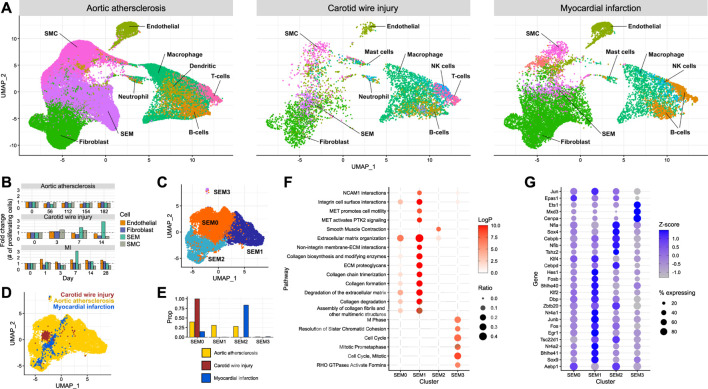
Comparison of SEM cells arising from different tissues **(A)** Integration of scRNA-seq datasets from carotid artery wire injury, aortic atherosclerosis and myocardial infarction. **(B)** Proportion of proliferating cells of each non-immune cell type across the time course of each scRNA-seq dataset. **(C)** Re-clustering of SEM cells identified across each of the three datasets, stratified by cluster. **(D)** Re-clustering of SEM cells identified across each of the three datasets, stratified by dataset of origin.**(E)** Proportions of SEM cells in each dataset assigned to each SEM cell cluster. **(F)** Pathway enrichment analysis of each SEM cell cluster. **(G)** Differentially expressed transcription factor genes in each SEM cell cluster.

Establishing the effect of different modes of injury on SEM cell expansion and their function in cardiovascular injury required the integration of multiple scRNA-seq datasets. Carotid injury data were integrated with data generated from aortic atherosclerosis (Pan et al., 2020) and myocardial infarction (Tombor et al., 2021). Using the aforementioned SEM cell marker genes along with other typical marker gene sets, cell types were assigned in each dataset (Figure 3A). SEM cells could be assigned in each dataset. They overlapped in the UMAP space, and were most abundant in the aortic atherosclerosis data. When assigning cell cycle scores to cells in each of the datasets, SEM cells displayed the greatest changes in proliferative state of non-immune cell types in response to the most acute modes of injury (carotid wire injury and myocardial infarction) (Figure 3B). This observation suggests that SEM cell expansion is induced by acute insults, whereas in the more chronic disease model of aortic atherosclerosis, few changes in proliferative dynamics could be detected amongst any non-immune cell population.

To identify potential variation within the SEM cell population, the cells were re-clustered (Figure 3C), resulting in four subpopulations. When stratifying the four SEM cell clusters identified across the datasets, it could be seen that carotid SEM cells almost exclusively occupied one cluster (SEM0) (Figures 3D, E **)**. Cardiac SEM cells were predominantly assigned to SEM2, whilst aortic SEM cells were split evenly across the major SEM clusters.

To gain further understanding into the prospective phenotypes of each SEM cell cluster, gene set enrichment analysis was performed using marker genes of each cluster. The SEM0 cluster exhibited the least concrete pathway enrichment (Figure 3F), perhaps indicating a less differentiated cellular state in comparison to the other clusters. SEM1 displayed a strong enrichment of pathways connected to synthesis and deposition of extracellular matrix, whereas SEM2 was the only cluster enriched for smooth muscle cell contraction. SEM3 - the smallest cluster - seemed to be a highly proliferative cell population. Cluster-specific pathway enrichment was supported by cluster-specific transcription factor gene expression (Figure 3G). The most proliferative cells were enriched in *Cenpa* expression - a transcription factor known to maintain proliferative potential (Swartz et al., 2019). Meanwhile, the SEM1 cluster expressed the highest levels of *Klf4*, a transcription factor linked to phenotypic modulation of smooth muscle cells which promotes extracellular matrix production (Shankman et al., 2015).

These data suggest that SEM cell phenotypes are determined by induction of individual transcriptional programs. Whether these are a consequence of the different tissue types, embryonic origin of the cell or the type of injury remains to be determined.

### 3.5 Modulated smooth muscle cells partially populate the neointima following injury

**FIGURE 4:**
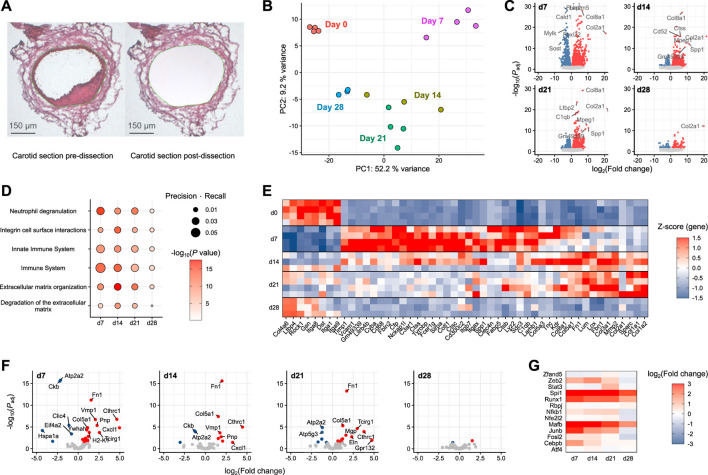
MACE-sequencing of neointimal tissue isolated from injured carotid arteries **(A)** Representative carotid section pre- and post-laser-capture microdissection (dissected area marked in green). **(B)** Principal component analysis of pooled neointima samples (*n* = 3-4) from pre-wire injury (Day 0) and days 7, 14, 21 and 28 post-injury. **(C)** Volcano plots of each day post-injury with differentially expressed genes highlighted in either blue (downregulated) or red (upregulated). **(D)** Pathway enrichment analysis of differentially expressed genes at each time point post-injury *versus* day 0 (pre-injury). **(E)** Top 50 differentially expressed genes assigned to significantly enriched pathways. **(F)** Volcano plots of SEM cluster marker genes as identified in scRNA-seq. **(G)** SEM-enriched transcription factor gene log fold changes in carotid neointima on days 7, 14, 21 and 28 post-wire injury *versus* day 0 (pre-injury).

A particular focus of the present study is neointima formation, a key factor in the pathology of vascular restenosis. Spatial information is missing from conventional scRNA-seq, and even spatial transcriptomics still lacks the resolution to deconvolute the very small neointima of the carotid artery. To determine whether SEM cells are involved in neointima formation, neointimal tissue from injured carotid arteries was captured by laser capture microdissection (Figure 4A) and subjected to gene expression profiling using nanoMACE-sequencing.

Covering a time frame from pre-injury to 4 weeks post-injury, the stages of acute inflammation, resolution and remodeling captured in the previously described single-cell RNA-sequencing data could be recapitulated using this approach. Compared to the naive vessel, the most dramatic alterations in gene expression were those observed at day 7 post-injury (Figures 4B, C). The day 7 samples were furthest removed from those at day 0 in a principal component analysis (Figure 4B), and the greatest number of differentially expressed genes was also detected at this time point (Figure 4C). Gene set enrichment analysis showed that this early-induced gene set was enriched for immune pathways relative to the other time points, specifically *Neutrophil degranulation*, *Innate immune system* and *Immune system* (Figure 4D). Subsequent time points (days 14 and 21) were enriched more so for pathways reflecting extracellular matrix organisation. By day 28, pathway enrichment was less evident and tissue more closely related to naive carotid tissue, suggesting the healing response had progressed towards resolution. The gene expression changes underlying these pathway enrichments can be clearly tracked across each time point after carotid injury (Figure 4E).

To examine the potential involvement of SEM cells in these processes, neointimal expression changes of marker genes identified in the single-cell RNA-sequencing data were examined. The strongest SEM cell signature was present at day 7 post-injury (Figure 4F), where the expression of most SEM cell marker genes were significantly upregulated. The signature was diminished across subsequent time points, and was almost absent by 28 days post-injury. This pattern was supported by changes in gene expression of SEM-enriched transcription factors identified in the previous analyses (Figure 4G). The strongest upregulation of these transcription factor genes was at 7 days post-injury, matching the time point at which the SEM cluster first appeared in the single-cell sequencing data.

Taken together, these data suggest that SEM cells have a presence in the neointima in the time period after acute carotid injury. Whether they are of importance for neointima formation is more difficult to establish, although given the aforementioned proliferative capacity of these cells *versus* other cells present in carotid tissue, they are certainly a candidate for further study.

## 4 Discussion

In this study, we describe gene expression profiles in the course of vascular injury on single cell level, as well as reporting transcriptional alterations in the neointima. We captured the process of wire-induced vascular injury using a time-resolved, single-cell approach. Moreover, we were able to identify a candidate transitional cell type which may play a key role in the physiological response to acute vascular injury.

The molecular mechanisms of restenosis have been studied for more than 3 decades. In large animals, oversized ballon-injury applied as a one or two hit model is the most frequently used technique. Given the small size of the murine carotid artery, balloon models are not feasible in mice (Pires et al., 2006; Ebert et al., 2021). As alternatives, other modes of vascular injury were developed, such as wire injury or electro injury models (Lindner et al., 1993; Yin et al., 2010). In C57BL/6 mice, superficial wire injury or electro-injury resulting in endothelial “denudation” only stimulates endothelial regrowth, but not smooth muscle cell proliferation. Contrastingly, the deep wire injury model applied in the present study disrupts the laminar elastica internal, and therefore elicits a strong healing reaction (Curaj et al., 2020). Disadvantages of the model include high variability and post-procedure thrombosis. In the present study, vessels with visually apparent thrombosis were excluded from the analysis.

Wire-induced carotid injury leads to neointima formation, smooth muscle cell dedifferentiation and endothelial denudation. The heterogeneous nature of the cell types and physiological processes involved makes the study of vascular injury highly complex. After the onset of injury, the vessel traverses several stages: acute inflammation, resolution of said inflammation and subsequent remodeling. The inflammatory phase is well described, but the exact process of resolution and its drivers remains unclear (Wu et al., 2017).

A variety of cell types are involved in the progression and resolution of inflammation following vascular injury. Throughout, cells display strong heterogeneity and plasticity. Single-cell RNA-sequencing is a powerful tool capable of capturing this heterogeneity (van Kuijk et al., 2019). Studies by Dobnikar et al. (2018) described vascular smooth muscle cell heterogeneity on single cell level in healthy tissue and atherosclerotic plaques. In these, *Sca1* upregulation was associated with phenotypic switching of vascular smooth muscle cells (Dobnikar et al., 2018). Kalluri et al. (2019) and Gu et al. (2019) described heterogeneity of aortic and adventitial tissue, and created an aortic atlas. Heterogeneity is already evident in healthy tissue, but it is further increased in disease. Heterogeneity in the context of atherosclerosis has been studied in *Ldlr* knockout mice (Cochain et al., 2018), and resulted in the identification of novel leukocyte (Winkels et al., 2018) and smooth muscle cell (Chappell et al., 2016) clusters.

The data presented herein collectively demonstrate the power of single-cell resolution data in the context of heterogeneity. Our study recapitulates the process of vascular injury and healing. We show differences in cellularity between control tissue and subsequent time points post-injury. Time-resolved data at single-cell resolution helps to identify activated cell populations responsible for vascular re-population following acute injury. Integration with further single-cell data enabled the identification of a previously identified form of modulated smooth muscle cell, which expands in a time-specific manner following injury - SEM cells. Data integration also permitted the comparison of SEM cells between different modes of injury, ranging from highly acute to chronic. In order to specifically study differences in smooth muscle cell clusters in disease, a smooth muscle cell-specific lineage tracing approach - as described by Alencar et al. (2020) and Dobnikar et al. (2018) - in combination with single-cell sequencing in healthy and injured tissue should be performed. Both studies provide insights into medial smooth muscle cell diversity. This approach could help to identify smooth muscle cell subpopulations, alongside determining the functional relevance of phenotypic changes in the resolution of disease. It could be shown that SEM cell expansion and phenotypes are likely driven by specific patterns of transcription factor expression and action. If this profile were unique to SEM cells, it may provide an avenue for therapeutic interventions targeting and moderating the response to vascular injury. Also required for this would be robust immunohistochemistry-based methods which would permit SEM cell identification based on protein expression rather than the transcriptome-based approach described here. This would allow a definitive conclusion on SEM cell localisation during restenosis.

In the course of this study, we describe different time points of vascular injury at both single-cell and neointima-specific levels. Both experiments enhanced understanding of inflammatory processes following vascular injury and revealed the importance of smooth muscle cell dedifferentiation to the resolution of inflammation and subsequent remodeling of carotid tissue.

## Data Availability

The datasets presented in this study can be found in online repositories. The names of the repository/repositories and accession number(s) can be found below: https://www.ncbi.nlm.nih.gov/geo/, GSE220514.
