# Supplementary material for: Acute injury to the mouse carotid artery provokes a distinct healing response
Source: Front Physiol. 2023 Feb 7;14:1125864. doi: 10.3389/fphys.2023.1125864 (PMC9941170; doi:10.3389/fphys.2023.1125864)
Supplement: Supplementary file 1 [file DataSheet1.PDF]

Supplementary Figure 1

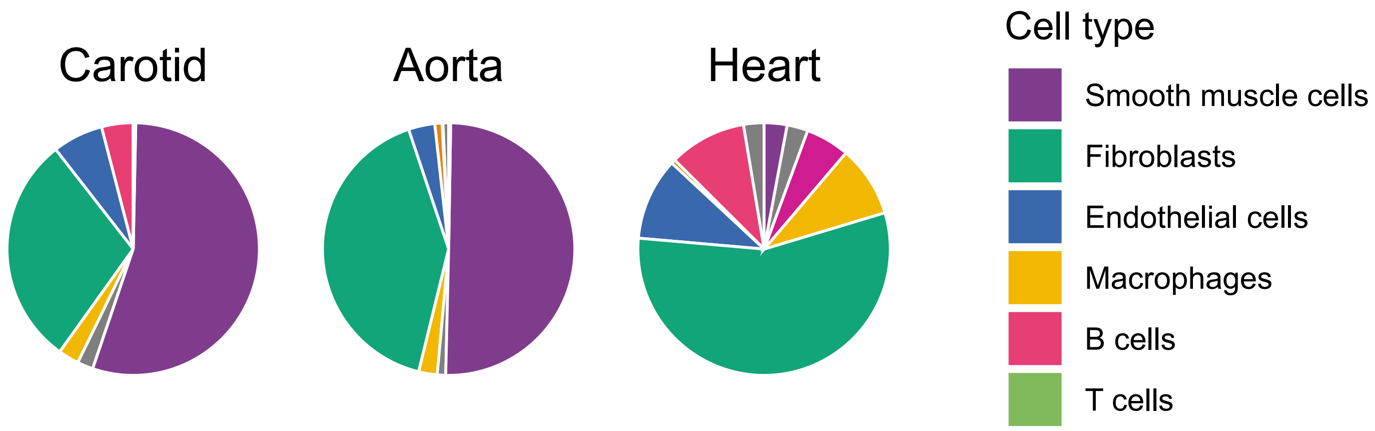

Supplementary Figure 1 | Cell type proportions in uninjured carotid, aortic and cardiac tissues
Proportion of each cell type present in scRNA-seq of uninjured carotid, aortic and cardiac tissues.

Supplementary Figure 2

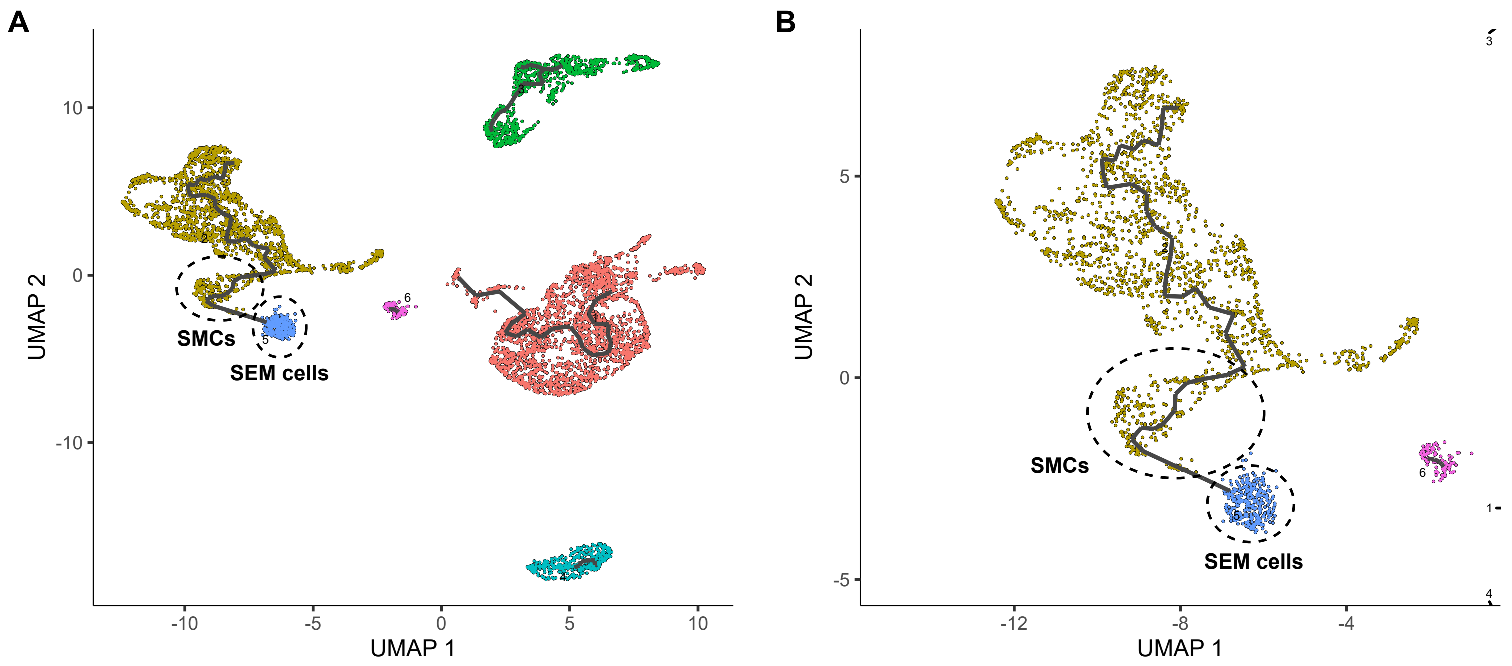

Supplementary Figure 2 | Trajectory analysis of carotid injury (A) Trajectories (black) built by *monocle3* over single cells present in the response to acute carotid injury. **(B)** Trajectory present between smooth muscle cell and SEM cell populations.

Supplementary Figure 3

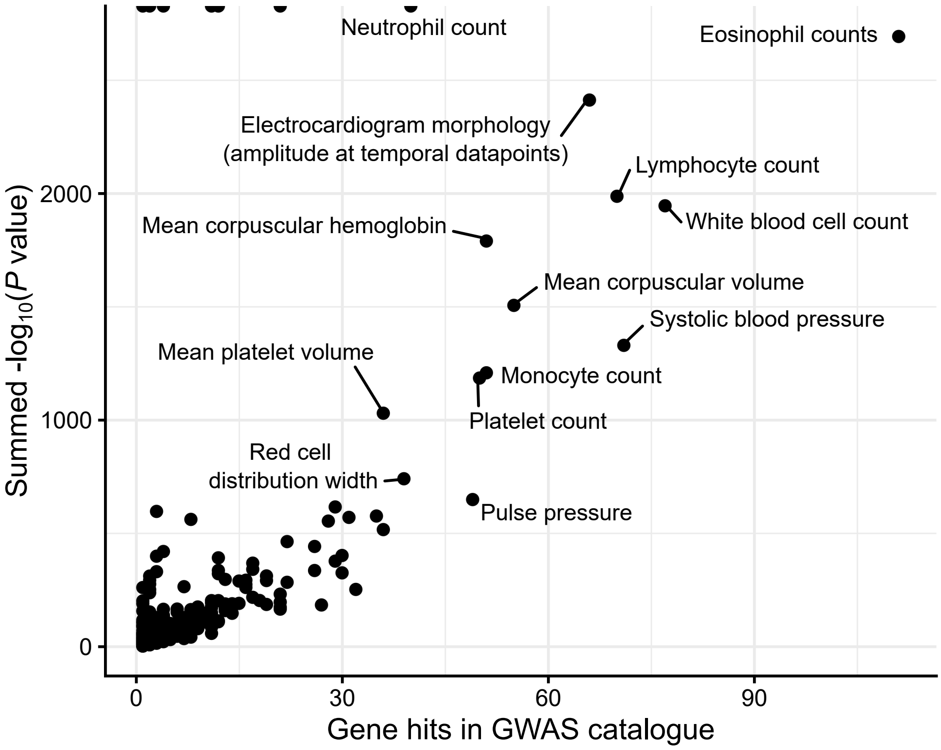

Supplementary Figure 3 | Association of SEM marker genes to human traits. Human homologues of SEM marker genes identified in mice were used to query the NHGRI-EBI GWAS Catalogue, and resutling hits were stratified by total genes assigned and the sum of log-transformed ***P*** values across all hits.
